# The impact of anastomotic leakage characteristics on the occurrence of anastomotic stenosis after colorectal resection, a retrospective cohort study

**DOI:** 10.1007/s00384-024-04699-4

**Published:** 2024-08-06

**Authors:** Schaima Abdelhadi, Emmanouil Tzatzarakis, Maike Hermann, Vanessa Orth, Katharina Vedder, Jannis Briscoe, Christoph Reissfelder, Flavius Șandra-Petrescu

**Affiliations:** 1 Department of Surgery, Medical Faculty Mannheim, Universitätsmedizin Mannheim, Heidelberg University, Theodor-Kutzer-Ufer 1-3, 68167 Mannheim, Germany; 2 DKFZ Hector Cancer Institute at the University Medical Center, Mannheim, Germany

**Keywords:** Anastomotic leakage, Anastomotic stenosis, Colorectal surgery, ISREC, ENPT

## Abstract

**Introduction:**

Anastomotic stenosis (AS) is a common complication after colorectal resection. However, the predisposing factors for stricture formation are not fully understood. Previous studies have shown anastomotic leakage (AL) to be a risk factor for the occurrence of AS. Therefore, we aim to investigate the impact of anastomotic leakage characteristics on the occurrence of anastomotic stenosis after colorectal resection.

**Methods:**

Consecutive patients with AL following elective, sphincter preserving, colorectal resection, with or without diversion ostomy, between January 2009 and March 2023 were identified from a prospectively collected database. The characteristics of the anastomotic leakage, patient baseline and operative characteristics as well as the postoperative outcomes were analyzed using univariate and multivariate logistic regression to identify factors associated with the occurrence of post-leakage AS.

**Results:**

A total of 129 patients developed AL and met the inclusion criteria. Among these, 28 (21.7%) patients were diagnosed with post-leakage AS. There was a significantly higher frequency of patients with neoadjuvant radiotherapy (18% vs 3%;* p* = .026) and hand-sewn anastomoses (39% vs 17%; *p* = .011) within the AS group. Furthermore, the extent of the anastomotic defect was significantly higher in the AS group compared with the non-AS group (50%, IQR 27–71 vs. 20%, IQR 9–40, *p* = 0.011). Similar findings were observed between the study groups regarding age, sex, BMI, ASA score, medical comorbidities, diagnosis, surgical procedure, surgical approach (open vs. minimally invasive), and anastomotic fashioning (side-to-end vs. end-to-end). On multivariate analysis, the extent of the anastomotic defect (OR 1.01; 95% CI 1.00–1.03; *p* = 0.034) and hand-sewn anastomoses (OR 2.68; 95% CI 1.01–6.98; *p* = 0.043) were confirmed as independent risk factors for post-leakage AS. No correlation could be observed between the occurrence of post-leakage AS and the ISREC grading of AL, the anastomotic height or the management of AL. Time to ostomy reversal was significantly longer in the AS group (202d, IQR 169–275 vs. 318d IQR 192–416, *p* = 0.014).

**Conclusion:**

The extent of the anastomotic defect and hand-sewn anastomoses were confirmed as independent risk factors for the occurrence of post-leakage AS. No correlation could be observed between the ISREC grading of AL, the anastomotic height or AL management, and the occurrence of post-leakage AS.

**Supplementary Information:**

The online version contains supplementary material available at 10.1007/s00384-024-04699-4.

## Introduction

The occurrence of anastomotic stenosis (AS) after colorectal surgery is a common complication, with reported incidences ranging from 2 to 28% [1–6]. The factors associated with the occurrence of AS remain not fully understood. Previous studies have identified radio- and chemotherapy, tissue perfusion at the anastomotic site, low rectal anastomosis, and anastomotic leakage (AL) as associated risk factors [2, 7, 8]. Among these factors, AL is a life-threatening complication with an incidence up to 20% [9, 10]. Management of AL has improved in the last decade, with endoscopic negative pressure therapy (ENPT) emerging as an important approach to preserve the anastomosis and reduce the rate of permanent ostomy [7, 11, 12].

However, the impact of anastomosis preservation on the occurrence of AS (i.e., post-leakage AS) in relation to the characteristics of the leakage is not yet fully understood. The definition and grading score of AL proposed in 2010 by the International Study Groups of Rectal Cancer (ISREC) as well as previous studies of Italian, Dutch, and Chinese colorectal study groups on AL, provide no information about the outcome of anastomotic preservation on the occurrence of post-leakage AS [13–15]. AS was shown to be associated with a reduced quality of life and an increased burden due to additional therapies such as endoscopic balloon dilation or surgical interventions [5]. The objective of the present study was therefore to investigate the impact of anastomotic leakage characteristics on the occurrence of anastomotic stenosis after colorectal resection.

## Methods

### Patient cohort and study design

Consecutive patients who underwent colorectal surgery between January 1, 2009, and March 15, 2023, at the Department of Surgery and Interdisciplinary Endoscopy, University Hospital Mannheim, Heidelberg University, were assessed for eligibility. Patients who were aged 18 years or older and underwent elective, sphincter preserving colorectal resection (anterior resection, low anterior resection, or sigmoid resection), with or without diversion ostomy, for benign or malignant disease were identified. Only patients who developed anastomotic leakage as defined by the International Study Group of Rectal Cancer (ISREC grade A/B/C) and who experienced no tumor relapse were included for analysis [13]. Exclusion criteria were patients with an inflammatory bowel disease, ileal pouch-anal anastomosis, multivisceral, and emergency resection due to perforation or bowel obstruction. This cohort study was conducted in line with the STROBE (Strengthening the Reporting of Observational studies in Epidemiology) guidelines and approved by the 2nd ethics committee at Heidelberg University (2019-826R) [16].

### Definitions and data acquisition

Clinicopathologic and postoperative data were extracted from prospectively maintained databases [11, 17, 18]. Anastomotic leakage was defined according to the ISREC grading [13]. The extent of the anastomotic defect was calculated as the size of the intestinal wall defect in relation to the anastomotic circumference. Anastomotic height was defined as the distance between the anastomosis and the anal verge and was obtained from endoscopic reports. Clinically relevant post-leakage AS was defined as the presence of endoscopically confirmed stricture at the site of the anastomosis, through which a standard-sized 9-mm endoscope cannot pass, and which occurred after successful treatment of AL. An impaired bowel function, with or without distention of the oral bowel, was mandatory.

Patients were grouped according to the occurrence of a clinically relevant post-leakage AS into two groups: AS group vs. non-AS group. The baseline and operative characteristic, postoperative outcomes, and characteristics of AL were further analyzed and compared between the groups.

### Perioperative and surgical care

Surgery was performed either minimally invasive or open based on patients’ performance status and preference, tumor characteristics (location and size), and surgeons’ discretion. All surgeries were performed by experienced attending colorectal surgeons. Perioperative care was identical in this cohort study and according to the local standard of care following a well-established multidisciplinary protocol as described in detail elsewhere [11, 17–20]. In summary, all patients who were suspected of having AL received routine endoscopic control. The endoscopic control was preferred to CT scan, as it provides a direct assessment of the AL and allows a contemporaneous treatment by ENPT. AL was clinically suspected based on clinical deterioration, increase of serum inflammatory markers such as C-reactive protein (CRP) or CRP in combination with leukocytosis and/or abnormal drain production [11]. Clinical deterioration was defined by a combination of tachycardia (> 100 beats per minute), tachypnea (ventilation rate of > 20 per minute), hypotension (systolic pressure < 100 mmHg), fever (> 38.5 °C), or progressive abdominal pain. Serum CRP levels of > 140 mg/dL were considered elevated. In the case of AL the extent of the anastomotic defect was reported in relation to the anastomotic circumference. Decision regarding the management of AL was made interdisciplinary while taking into consideration the clinical condition of the patient, the extent of the anastomotic defect and leakage cavity, the leakage distance to the anal verge, and the blood supply at the anastomotic site. The algorithm of AL management including a step-up approach in case of therapy failure was previously described [11]. In summary, the endoscopic therapy consisted of cavity rinse using irrigation and ENPT. The ENPT system consisted of an endo-sponge that was connected with a negative pressure system. The sponge had to fit into the leakage cavity, in order to close it completely when negative pressure was applied, and should not extend to the lumen. Thus, the entire cavity is drained and continuously downsized. Endoscopic changes were performed at the earliest after 3–4 days but no later than 7 days, or in case of vacuum loss or sponge dislocation. The therapy was stopped as soon as the leakage cavity was completely closed or completely covered with healthy granulation tissue [11]. However, if a closure of the leakage cavity cannot be accomplished by ENPT, or in case of clinical deterioration under ENPT, surgical revision (i.e., anastomotic repair or redo) or even Hartmann’s procedure was considered.

After successful AL management, a further endoscopic control was obligatory for every anastomosis, prior to the ostomy reversal or in case of impaired bowel function (e.g., constipation, need to push, incontinence, tenesmus, ribbon stools).

### Statistical analysis

The statistical analysis was performed using SPSS Premium (IBM SPSS Statistics 27©). Categorical parameters are expressed as frequencies and were compared using the Pearson *χ*^2^ test or Fisher exact test. Continuous variables are reported as mean (SD) or median (IQR), depending on the distribution pattern, and were compared using the two-tailed t test or Mann–Whitney test. Univariate and multivariate logistic regression analyses were performed to identify factors associated with the occurrence of post-leakage AS. *P*-values < 0.05 were defined as statistically significant.

## Results

**Fig. 1:**
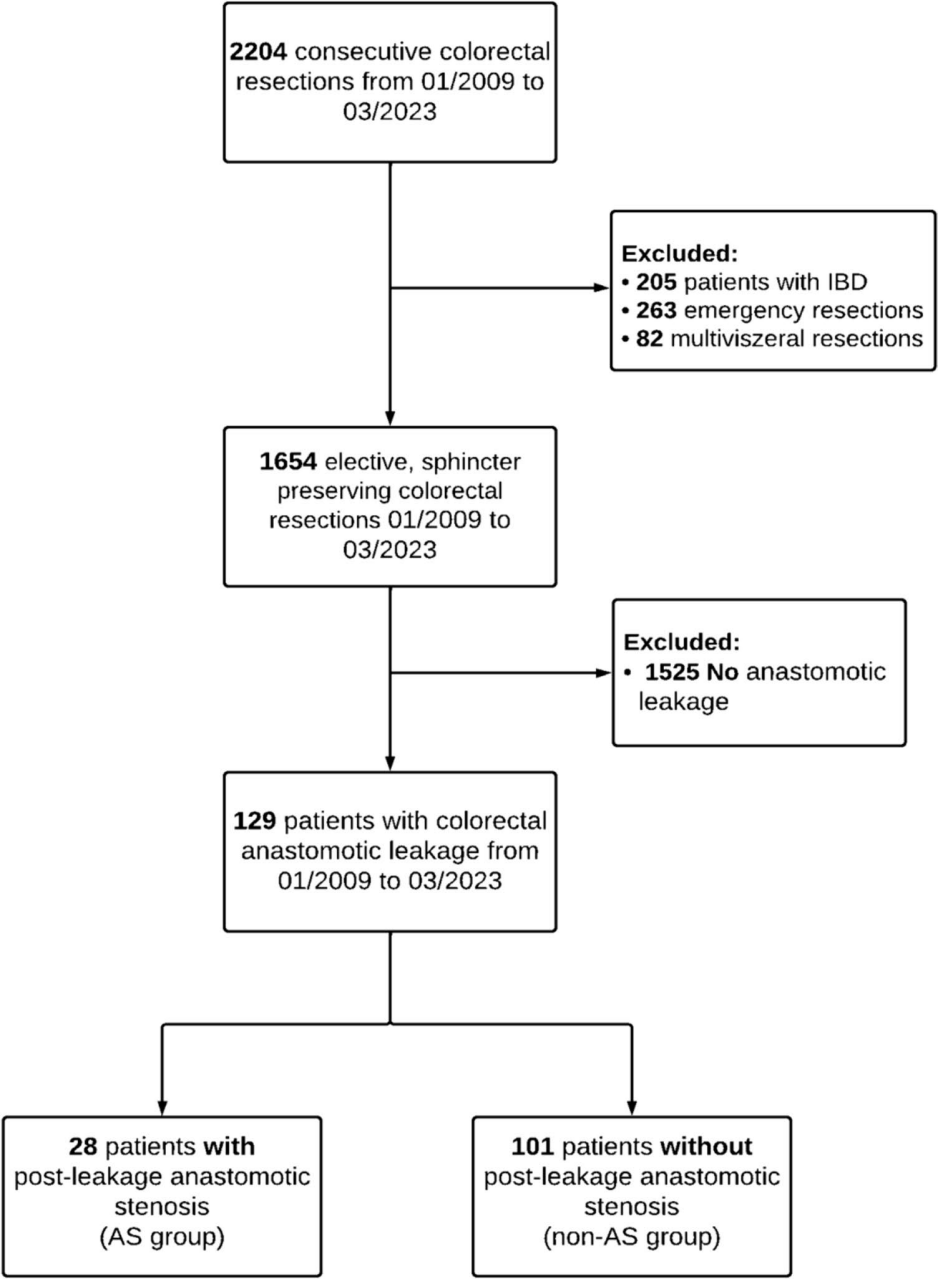
Patient flowchart

A total of 2204 consecutive patients with colorectal resection, for benign or malignant disease, were screened for eligibility, of whom 129 patients (90 [70%] men; 39 [30%] women) developed AL and met the inclusion criteria. Among these, 28 patients (22%) developed a post-leakage AS (AS group) and 101 (78%) did not (non-AS group) (Fig. 1). The median follow-up period for the study population was 979 days (IQR 397–1993).

### Patient characteristics

Patient demographic and clinical characteristics are detailed in Table 1. There was a significantly higher frequency of patients with pervious neoadjuvant radiotherapy (18% vs 3%; *p* = 0.026) in the AS group compared with the non-AS group, while the other baseline characteristics were well-balanced between the study groups and yielded no statistical significance regarding age, sex, BMI, ASA score, medical comorbidities, diagnosis, or adjuvant therapy (Table 1).

**Table 1:** Baseline characteristics

Characteristics	Non-AS group (*n* = 101)	AS group (*n* = 28)	*p*-value
Age, years^a^	62 (54–71)	64 (56–70)	0.782
BMI, kg/m^2a^	26 (23–30)	25 (23–28)	0.855
Sex ratio, male: female	72:29	18:10	0.475
ASA			0.935
I	13 (13)	4 (14)	
II	58 (57)	15 (54)	
III	30 (30)	9 (32)	
IV	0 (0)	0 (0)	
Cardiovascular comorbidities	59 (58)	18 (64)	0.575
Diabetes mellitus	13 (13)	4 (14)	0.845
Pulmonary comorbidities	20 (19)	5 (18)	0.818
Diagnosis			0.624
CRC	85 (84)	25 (89)	
Diverticulitis	9 (9)	3 (11)	
Rectal prolapse	3 (3)	0 (0)	
Other malignant lesions	4 (4)	0 (0)	
Neoadjuvant treatment			***0.026***
Neoadjuvant radiochemotherapy	33 (33)	10 (36)	
Neoadjuvant radiotherapy	3 (3)	5 (18)	
Neoadjuvant chemotherapy	5 (5)	1 (4)	
Adjuvant treatment	24 (24)	10 (36)	0.102

*BMI* body mass index, *ASA* American Society of Anesthesiologists, *CRC* colorectal cancer

^a^Values are median (interquartile range)

### Operative characteristics and postoperative outcomes

Operative characteristics and postoperative outcomes are presented in Table 2. Similar findings were observed between the study groups concerning the surgical procedure, the surgical approach (open vs. minimally invasive), and anastomotic fashioning (side-to-end vs. end-to-end). The majority of patients in both groups underwent a minimally invasive approach (non-AS: 70% vs AS: 64%, *p* = 0.543). Low anterior rectum resection (LAR) was the most frequently performed surgical procedure in both groups (non-AS: 70% vs AS: 75%, *p* = 0.888). In the non-AS group, 71% of the patients received a side-to-end anastomosis, while in the AS group, 54% received an end-to-end anastomosis (*p* = 0.081). Notably, there was a significantly higher frequency of patients with hand-sewn anastomoses (39% vs 17%; *p* = 0.011) in the AS group compared with the non-AS group.

**Table 2:** Operative characteristics and postoperative outcomes

Characteristics	Non-AS group (*n* = 101)	AS group (*n* = 28)	*p*-value
Surgical procedure			0.888
AR	13 (13)	3 (11)	
LAR	71 (70)	21 (75)	
Sigmoid resection	17 (16)	4 (14)	
Protective ostomy	76 (75)	21 (75)	0.979
Surgical approach			0.543
Minimally invasive	71 (70)	18 (64)	
Open	30 (30)	10 (36)	
Anastomotic fashioning			0.081
S–E	72 (71)	15 (54)	
E–E	29 (29)	13 (46)	
Anastomotic technique			***0.011***
Stapler	84 (83)	17 (61)	
Hand sewn	17 (17)	11 (39)	

*AR* anterior resection; *LAR* low anterior resection; *S-E* side-to-end; *E-E* end-to-end

### Characteristics and management of AL

Characteristics and management of AL are presented in Table 3. The comparisons of the ISREC grading of AL revealed similar findings between both groups (*p* = 0.216). Furthermore, the postoperative day (POD) of AL diagnosis and the anastomotic height were similar in both groups. Interestingly, the extent of the anastomotic defect was significantly higher in the AS group compared with the non-AS group (50%, IQR 27–71 vs. 20%, IQR 9–40, *p* = 0.011). AL management within both groups was carried out mostly by ENPT (60% vs. 60%, *p* = 0.832), in addition to antibiotic therapy (83% vs. 86%, *p* = 0.897). The median number of ENPT cycles was similar in both groups (non-AS: 4, IQR 2–6 vs. AS: 5 IQR 3–7, *p* = 0.774). Forty-three of the patients in the AS group and 34% of the patients in the non-AS group underwent surgical revision (*p* = 0.911). Among these, 73% underwent peritoneal wash-out, 55% anastomotic oversewing, and 42% required ENPT additional to surgical revision. No differences were noticed in the AL management and the length of hospital stay (non-AS: 20d IQR 14–35 vs. AS: 23d IQR 16–43, *p* = 0.297) in both groups. The ostomy could be reversed significantly earlier in the non-AS group (202d, IQR 169–275 vs. 318d IQR 192–416,* p* = 0.014). Both groups had a similar rate of re-ostomy (non-AS 33% vs. AS 36%; *p* = 0.841) and reversal of the re-ostomy (non-AS: 30% vs. AS: 10%; *p* = 0.076).

**Table 3:** Characteristics and management of AL

Characteristics	Non-AS group (*n* = 101)	AS group (*n* = 28)	*p*-value
Anastomotic leakage^a^			0.316
Grade A	16 (16)	2 (7)	
Grade B	51 (50)	15 (54)	
Grade C	34 (34)	11 (39)	
POD of AL diagnosis, d^b^	7 (6–13)	7 (5–16)	0.861
Anastomotic height^b^	6 (4–9)	5 (4–7)	0.456
≤ 5 cm	47 (46)	14 (50)	
6–10 cm	38 (38)	9 (32)	
> 10 cm	16 (16)	5 (18)	
Extent of the anastomotic defect^b^	20 (9–40)	50 (27–71)	***0.011***
Up to 24%	71 (70)	14 (50)	
25–49%	18 (18)	9 (32)	
50–74%	8 (8)	4 (14)	
75–100%	4 (4)	1 (4)	
Management of AL			
ENPT	59 (60)	17 (60)	0.832
Cycles^b^	4 (2–6)	5 (3–7)	0.774
Surgical revision*	34 (34)	11 (39)	0.911
Antibiotic therapy	84 (83)	24 (86)	0.897
LOS, d^b^	20 (14–35)	23 (16–43)	0.297
Ostomy reversal	74 (73)	22 (78)	0.576
POD of ostomy reversal, d^b^	202 (169–275)	318 (192–416)	***0.014***
Re-ostomy	34 (33)	10 (36)	0.841
Reversal of the re-ostomy	10 (30)	1 (10)	0.076

*POD* postoperative day; *AL* anastomotic leakage; *ENPT* endoscopic negative pressure therapy; *LOS* length of stay;* d* day

^a^As defined by the International Study Group of Rectal Cancer

^b^Values are median (interquartile range)

*Eight patients in the non-AS and two in the AS group underwent ENPT additionally to surgical revision

Most anastomotic strictures (*n* = 124, 96%) were treated by endoscopic balloon dilation with a success rate of 85%. A deviating ostomy was formed in three patients while a permanent ostomy was necessary in one patient. Anastomosis resection was necessary in two patients.

### Risk factors for post-leakage AS

To further scrutinize potential factors associated with the occurrence of post-leakage AS a logistic regression analysis was performed. Univariate analysis of these factors found that neoadjuvant radiotherapy (odds ratio [OR] 9.13; 95% CI, 2.15–48.48; *p* = 0.004), hand-sewn anastomoses (OR 2.99; 95% CI 1.16–7.60; *p* = 0.022), and the extent of the anastomotic defect (OR 1.02; 95% CI 1.00–1.03; *p* = 0.011) were associated with occurrence of post-leakage AS (Supplement 1). On multivariate analysis, the extent of the anastomotic defect (OR 1.01; 95% CI 1.00–1.03; *p* = 0.034) and hand-sewn anastomoses (OR 2.68; 95% CI 1.01–6.98; *p* = 0.043) were confirmed as independent risk factors for post-leakage AS (Table 4).

**Table 4:** Multivariate regression analysis of factors associated with post-leakage AS

Characteristics	OR	95%CI	*p*-value
Neoadjuvant treatment			
Neoadjuvant radiotherapy (yes vs. no)	0.71	0.29–1.73	0.445
Anastomotic technique			
Stapler vs. hand sewn	2.68	1.01–6.98	***0.043***
Extent of the anastomotic defect	1.01	1.00–1.03	***0.034***

*OR* odds ratio*, CI* confidence interval

## Discussions

Of the present study population (*n* = 129), a total of 28 patients (21.7%) developed post-leakage AS. Previous studies have reported an overall rate of AS after colorectal surgery of up to 30% [2–4, 21]. However, none of these studies specifically investigated the subgroup of patients who developed post-leakage AS after successful treatment of AL. Since AL was previously shown to be a risk factor for the occurrence of AS, and the present study specifically investigated this subgroup of patients, it is expected that the rate of post-leakage AS within the group is high [2]. To the best of our knowledge, this is the first study to investigate the occurrence of AS in relation to the characteristics of AL.

Management of AL has improved in the last decades due to advances in therapy methods. Surgical procedures, such as anastomotic resection with re-anastomosis or Hartmann’s resection, have been replaced by less invasive approaches such as ENPT [7, 11, 12]. However, the outcome of a successful AL treatment with regard to the occurrence of AS has not been adequately investigated [2]. AS is a common, clinically relevant, complication after colorectal resection, associated with increased burden and reduced quality of life [5]. Its management requires invasive or non-invasive approaches, such as anastomotic resection with or without preservation of the intestinal continuity, new formation of a deviating or permanent ostomy, endoscopic dilation, or stapler stricturoplasty [18, 22].

In the present study, more than half of the patients in both groups underwent ENPT for the management of AL. Interestingly, neither ENPT per se, nor the number of ENPT cycles influenced the occurrence of post-leakage AS, which is similar to the results of other studies [3]. However, data regarding the rate of post-leakage AS after ENPT is scarce. The previous study of Bertocchi et al. identified protective ileostomy as a predictive factor for AS after rectosigmoid resection [23]. In contrast and similar to Chong et al., the present study revealed no influence of the primary ostomy on the occurrence of post-leakage AS [2]. The reversal rate of the primary formed ostomy was similar in both groups. Both groups showed a relatively long median time to ostomy reversal. This may be explained by the AL leading to a reduced clinical condition and nutritional status of the patients, requiring a longer recovery time [24]. Furthermore, adjuvant chemotherapy may contribute to a delayed ostomy reversal, as previous studies showed [24, 25]. In line with previous studies, post-leakage AS resulted in a significant delay in ostomy reversal compared to the non-AS group. It can be assumed that AS and its therapy-related morbidity negatively influenced the clinical condition of the patients, which is consistent with the findings of other studies [5]. While there is no consensus for the optimal timing of ostomy reversal after colorectal surgery, there is increasing interest in the concept of early reversal in suitable patients [26]. Several studies showed an increased risk for postoperative complications, particularly postoperative ileus, in cases of ostomy reversal 6 months or longer after the initial operation [24–27]. To determine the optimal timing of ostomy reversal in the context of adjuvant chemotherapy, our study group conducted the “CoCStom trial,” a randomized multicenter trial comparing completeness of adjuvant chemotherapy after early (8–10 days after LAR) versus late ostomy reversal (~ 26 weeks after LAR) in low anterior resection for rectal cancer [17]. Early results are promising and will be published soon.

Interestingly, the extent of the anastomotic defect was significantly higher in the AS group compared with the non-AS group. To the best of our knowledge, there are no published data about the correlation of the defect extension and the occurrence of post-leakage stenoses. The reason why leakages with a larger defect led to a higher stenosis rate is unclear. One may suppose that larger defects or leakage cavities lead to a stronger inflammatory reaction and fibrosis formation as the smaller ones. These stenoses would be classified as grade 2b and 3 according to Cong et al. [i.e., clinically relevant stricture with fibrotic adhesions at the anastomotic site which cannot be broken by digital examination, associated (grade 3) or not (grade 2b) with an upstream dilation] [2]. Inflammation and subsequently fibrosis were previously shown to be risk factors for occurrence of AS after resection for diverticulitis, endometriosis, or Crohn’s disease [5, 23, 28]. The ISREC grading of AL was previously proposed to simplify the characterization of AL according to the clinical condition of the patients and the applied therapy [13]. However, there are no previous investigations on correlation between post-leakage AS and the clinically relevant ISREC grades of AL (B and C). Since it was previously shown to be applicable in clinical studies, we used the ISREC classification and grading of AL for the first time in relation to post-leakage AS [11, 14, 29, 30]. Interestingly, a stratification according to ISREC grading score shows similar rates of post-leakage AS irrespectively of an endoscopic (i.e., grade B leakage) or a surgical approach (i.e., grade C leakage). This suggests that an endoscopic management of AL can be primarily considered, without an increased risk for anastomotic stenosis. Laborious re-surgery for AL may be therefore avoided and the intestinal continuity preserved.

Similar to Cong et al. and Surek et al., the distance from the anastomosis to the anal verge does not influence the rate of AS [2, 21]. Previous data is controversial. Lucha et al. found an increased risk for stricture in cases of anastomoses situated underneath the peritoneal reflection [31]. However, recent data from the Cochrane database show no influence of the anastomotic height on the rate of AS in relation to different anastomotic types [32]. The data reported in these studies include elective colorectal surgery irrespective of AL. Contrarily to previous studies, which showed less AS for hand-sewn anastomoses, our study identified hand-sewn anastomoses as an independent risk factor for post-leakage AS. However, no correlation could be observed between the type of anastomotic fashioning (E-E or S-E) and the occurrence of post-leakage AS, which is in line with a recent systematic review and network meta-analysis of 29 RCTs that showed similar rates of AS in both E-E and S-E anastomosis [33]. The index procedure did not influence the occurrence of post-leakage AS in the present study.

Similar to the findings of Surek et al. and Bertocchi et al., BMI was not a predisposing factor for post-leakage AS in the present study [21, 23]. This is interesting since it was previously shown that higher BMI values correlates with AL [21, 34].

The main limitation of the present study is given by the retrospective study design, with a potential selection and reporting bias. Moreover, there is no generally accepted definition of AS since different endoscopes or diagnostic methods are used by different endoscopists within the trials, which makes it difficult to compare the results between trials. Lastly, our results were from a single-center study with a relatively small sample size. Further confirmation of the results in prospective, observational studies in a multicenter setting are necessary, as prospective randomized trials are difficult to plan due to the nature of the disease. In order to reduce the selection bias, only clinically relevant AS were included in the present study, which are more likely to be generally accepted within trials.

## Conclusion

The extent of the anastomotic defect and hand-sewn anastomoses are independent risk factors for the occurrence of post-leakage AS. No correlation could be observed between the ISREC grading of AL, the anastomotic height, or AL management and the occurrence of post-leakage AS. Considering the retrospective nature of the study, the present results should be carefully interpreted. Further prospective, observational studies in a multicenter setting are necessary to confirm these results.

## Supplementary Information

Below is the link to the electronic supplementary material.

Supplement 1 Univariate Analysis of factors associated with post-leakage AS (PDF 104 KB)

## Data Availability

The data that support the findings of this study are available on request from the corresponding author.

## References

[1] Chan RH, Lin SC, Chen PC, Lin WT, Wu CH, Lee JC, Lin BW (2020) Management of colorectal anastomotic stricture with multidiameter balloon dilation: long-term results. Tech Coloproctol 24(12):1271–1276 32757156 10.1007/s10151-020-02318-2 PMC7661393

[2] Cong J, Zhang H, Chen C (2023) Definition and grading of anastomotic stricture/stenosis following low anastomosis after total mesorectal excision: a single-center study. Asian J Surg 46(9):3722–3726. 10.1016/j.asjsur.2023.03.027 10.1016/j.asjsur.2023.03.027 36967350

[3] Popivanov GI, Mutafchiyski VM, Cirocchi R, Chipeva SD, Vasilev VV, Kjossev KT, Tabakov MS (2020) Endoluminal negative pressure therapy in colorectal anastomotic leaks. Colorectal Dis 22(3):243–253 31274227 10.1111/codi.14754

[4] Schlegel RD, Dehni N, Parc R, Caplin S, Tiret E (2001) Results of reoperations in colorectal anastomotic strictures. Dis Colon Rectum 44(10):1464–1468 11598475 10.1007/BF02234598

[5] Polese L, Vecchiato M, Frigo AC, Sarzo G, Cadrobbi R, Rizzato R, Bressan A, Merigliano S (2012) Risk factors for colorectal anastomotic stenoses and their impact on quality of life: what are the lessons to learn? Colorectal Dis 14(3):e124-128 21910814 10.1111/j.1463-1318.2011.02819.x

[6] Luchtefeld MA, Milsom JW, Senagore A, Surrell JA, Mazier WP (1989) Colorectal anastomotic stenosis. Results of a survey of the ASCRS membership. Dis Colon Rectum 32(9):733–736 2667922 10.1007/BF02562119

[7] Adamenko O, Ferrari C, Seewald S, Schmidt J (2022) Prophylactic endoluminal vacuum therapy after major gastrointestinal surgery: a systematic review. Updates Surg 74(4):1177–1186 35262844 10.1007/s13304-022-01265-x

[8] Clifford RE, Fowler H, Manu N, Vimalachandran D (2021) Management of benign anastomotic strictures following rectal resection: a systematic review. Colorectal Dis 23(12):3090–3100 34374203 10.1111/codi.15865

[9] Rickert A, Willeke F, Kienle P, Post S (2010) Management and outcome of anastomotic leakage after colonic surgery. Colorectal Dis 12(10 Online):e216-223 20002697 10.1111/j.1463-1318.2009.02152.x

[10] Nesbakken A, Nygaard K, Lunde OC, Blucher J, Gjertsen O, Dullerud R (2005) Anastomotic leak following mesorectal excision for rectal cancer: true incidence and diagnostic challenges. Colorectal Dis 7(6):576–581 16232238 10.1111/j.1463-1318.2005.00870.x

[11] Sandra-Petrescu F, Tzatzarakis E, Kahler G, Reissfelder C, Herrle F (2021) Management of colorectal anastomotic leakage using endoscopic negative pressure therapy with or without protective ostomy: a retrospective study. Int J Colorectal Dis 36(10):2261–2269 34455472 10.1007/s00384-021-04011-8 PMC8426235

[12] Talboom K, Greijdanus NG, van Workum F, Ubels S, Rosman C, Hompes R, de Wilt JHW, Tanis PJ (2022) group TE-Rw: International expert opinion on optimal treatment of anastomotic leakage after rectal cancer resection: a case-vignette study. Int J Colorectal Dis 37(9):2049–2059 36002748 10.1007/s00384-022-04240-5 PMC9436864

[13] Rahbari NN, Weitz J, Hohenberger W, Heald RJ, Moran B, Ulrich A, Holm T, Wong WD, Tiret E, Moriya Y et al (2010) Definition and grading of anastomotic leakage following anterior resection of the rectum: a proposal by the International Study Group of Rectal Cancer. Surgery 147(3):339–351 20004450 10.1016/j.surg.2009.10.012

[14] Spinelli A, Anania G, Arezzo A, Berti S, Bianco F, Bianchi PP, De Giuli M, De Nardi P, de Paolis P, Foppa C et al (2020) Italian multi-society modified Delphi consensus on the definition and management of anastomotic leakage in colorectal surgery. Updat Surg 72(3):781–792 10.1007/s13304-020-00837-z 32613380

[15] van Rooijen SJ, Jongen AC, Wu ZQ, Ji JF, Slooter GD, Roumen RM, Bouvy ND (2017) Definition of colorectal anastomotic leakage: a consensus survey among Dutch and Chinese colorectal surgeons. World J Gastroenterol 23(33):6172–6180 28970733 10.3748/wjg.v23.i33.6172 PMC5597509

[16] von Elm E, Altman DG, Egger M, Pocock SJ, Gøtzsche PC, Vandenbroucke JP (2007) The Strengthening the Reporting of Observational Studies in Epidemiology (STROBE) statement: guidelines for reporting observational studies. PLoS Med 4(10):e296 17941714 10.1371/journal.pmed.0040296 PMC2020495

[17] Sandra-Petrescu F, Herrle F, Hinke A, Rossion I, Suelberg H, Post S, Hofheinz RD, Kienle P (2015) CoCStom trial: study protocol for a randomised trial comparing completeness of adjuvant chemotherapy after early versus late diverting stoma closure in low anterior resection for rectal cancer. BMC Cancer 15(1):923 26589718 10.1186/s12885-015-1838-0 PMC4654836

[18] Kouladouros K, Reissfelder C, Kahler G (2023) Endoscopic stricturoplasty with linear stapler: an efficient alternative for the refractory rectal anastomotic stricture. Dig Dis Sci 68(12):4432–4438 37855986 10.1007/s10620-023-08156-0 PMC10635923

[19] Seyfried S, Herrle F, Schröter M, Hardt J, Betzler A, Rahbari NN, Reißfelder C (2021) Initial experiences with the implementation of the enhanced recovery after surgery (ERAS®) protocol. Chirurg 92(5):428–433 33471183 10.1007/s00104-020-01341-1

[20] Galata C, Vassilev G, Haas F, Kienle P, Büttner S, Reißfelder C, Hardt J (2019) Clinical, oncological, and functional outcomes of Da Vinci (Xi)-assisted versus conventional laparoscopic resection for rectal cancer: a prospective, controlled cohort study of 51 consecutive cases. Int J Colorectal Dis 34(11):1907–1914 31642968 10.1007/s00384-019-03397-w

[21] Surek A, Donmez T, Gemici E, Dural AC, Akarsu C, Kaya A, Ferahman S, Bozkurt MA, Karabulut M, Alis H (2023) Risk factors affecting benign anastomotic stricture in anterior and low anterior resections for colorectal cancer: a single-center retrospective cohort study. Surg Endosc 37(7):5246–5255 36964291 10.1007/s00464-023-10002-3

[22] Kang J, Kim H, Park H, Lee B, Lee KY (2022) Risk factors and economic burden of postoperative anastomotic leakage related events in patients who underwent surgeries for colorectal cancer. PLoS One 17(5):e0267950 35584082 10.1371/journal.pone.0267950 PMC9116683

[23] Bertocchi E, Barugola G, Benini M, Bocus P, Rossini R, Ceccaroni M, Ruffo G (2019) Colorectal anastomotic stenosis: lessons learned after 1643 Colorectal Resections for Deep Infiltrating Endometriosis. J Minim Invasive Gynecol 26(1):100–104 29678755 10.1016/j.jmig.2018.03.033

[24] Waterland P, Goonetilleke K, Naumann DN, Sutcliff M, Soliman F (2015) Defunctioning ileostomy reversal rates and reasons for delayed reversal: does delay impact on complications of ileostomy reversal? A study of 170 defunctioning ileostomies. J Clin Med Res 7(9):685–689 26251682 10.14740/jocmr2150w PMC4522985

[25] Taylor D, Besson A, Faragher IG, Chan STF, Yeung JM (2021) Investigations and time trends in loop ileostomy reversals following anterior resections: a single Australian institution seven-years’ experience. ANZ J Surg 91(5):938–942 33300280 10.1111/ans.16483

[26] Khoo TW, Dudi-Venkata NN, Beh YZ, Bedrikovetski S, Kroon HM, Thomas ML, Sammour T (2021) Impact of timing of reversal of loop ileostomy on patient outcomes: a retrospective cohort study. Tech Coloproctol 25(11):1217–1224 34499279 10.1007/s10151-021-02516-6

[27] Rubio-Perez I, Leon M, Pastor D, Diaz Dominguez J, Cantero R (2014) Increased postoperative complications after protective ileostomy closure delay: an institutional study. World J Gastrointest Surg 6(9):169–174 25276286 10.4240/wjgs.v6.i9.169 PMC4176777

[28] Gordon IO, Bettenworth D, Bokemeyer A, Srivastava A, Rosty C, de Hertogh G, Robert ME, Valasek MA, Mao R, Li J et al (2022) International consensus to standardise histopathological scoring for small bowel strictures in Crohn’s disease. Gut 71(3):479–486 33952604 10.1136/gutjnl-2021-324374 PMC8903083

[29] Kulu Y, Ulrich A, Bruckner T, Contin P, Welsch T, Rahbari NN, Buchler MW, Weitz J (2013) International Study Group of Rectal C: validation of the International Study Group of Rectal Cancer definition and severity grading of anastomotic leakage. Surgery 153(6):753–761 23623834 10.1016/j.surg.2013.02.007

[30] Sandra-Petrescu F, Rahbari NN, Birgin E, Kouladouros K, Kienle P, Reissfelder C, Tzatzarakis E, Herrle F (2023) Management of anastomotic leakage after colorectal resection: survey among the German CHIR-Net Centers. J Clin Med 12(15):4933 37568336 10.3390/jcm12154933 PMC10419945

[31] Lucha PA Jr, Fticsar JE, Francis MJ (2005) The strictured anastomosis: successful treatment by corticosteroid injections–report of three cases and review of the literature. Dis Colon Rectum 48(4):862–865 15747075 10.1007/s10350-004-0838-y

[32] Neutzling CB, Lustosa SA, Proenca IM, da Silva EM, Matos D (2012) Stapled versus handsewn methods for colorectal anastomosis surgery. Cochrane Database Syst Rev 2:CD003144 10.1002/14651858.CD003144.pub2 PMC12009662 22336786

[33] Liu H, Xiong M, Zeng Y, Shi Y, Pei Z, Liao C (2023) Comparison of complications and bowel function among different reconstruction techniques after low anterior resection for rectal cancer: a systematic review and network meta-analysis. World J Surg Oncol 21(1):87 36899350 10.1186/s12957-023-02977-z PMC9999608

[34] Silva-Velazco J, Stocchi L, Costedio M, Gorgun E, Kessler H, Remzi FH (2016) Is there anything we can modify among factors associated with morbidity following elective laparoscopic sigmoidectomy for diverticulitis? Surg Endosc 30(8):3541–3551 26541732 10.1007/s00464-015-4651-6

